# Earthquake Detection in a Static and Dynamic Environment Using Supervised Machine Learning and a Novel Feature Extraction Method

**DOI:** 10.3390/s20030800

**Published:** 2020-02-01

**Authors:** Irshad Khan, Seonhwa Choi, Young-Woo Kwon

**Affiliations:** 1 School of Computer Science, Kyungpook National University, Daegu 41566, Korea; irshad.khaan@gmail.com; 2 National Disaster Management Research Institute, Ulsan 44538, Korea; shchoi33@korea.kr

**Keywords:** earthquake detection, time-series features, Internet of Things, machine learning

## Abstract

Detecting earthquakes using smartphones or IoT devices in real-time is an arduous and challenging task, not only because it is constrained with the hard real-time issue but also due to the similarity of earthquake signals and the non-earthquake signals (i.e., noise or other activities). Moreover, the variety of human activities also makes it more difficult when a smartphone is used as an earthquake detecting sensor. To that end, in this article, we leverage a machine learning technique with earthquake features rather than traditional seismic methods. First, we split the detection task into two categories including static environment and dynamic environment. Then, we experimentally evaluate different features and propose the most appropriate machine learning model and features for the static environment to tackle the issue of noisy components and detect earthquakes in real-time with less false alarm rates. The experimental result of the proposed model shows promising results not only on the given dataset but also on the unseen data pointing to the generalization characteristics of the model. Finally, we demonstrate that the proposed model can be also used in the dynamic environment if it is trained with different dataset.

## 1. Introduction

Due to the nature of earthquakes, significant research efforts have been made to develop real-time earthquake detection systems for disaster management. Earthquake fatal levels of motion can cause fatalities and damage in populated regions [1]. Because typical human structures are unable to resist large magnitude earthquakes, possible ways to overcome such fatalities are to build earthquake-resistant buildings or to take advantage of an Earthquake Early Warning (EEW) system that provides seconds to minutes of warning in advance, thereby allowing people to move to safe areas or shut down dangerous machinery. However, it is not only costly to construct earthquake-resistant structures but also difficult to build a highly accurate nationwide EEW system.

In recent years, emerging computing technologies such as mobile computing and Internet-of-Thing (IoT) systems equipped with various MEMS (Micro Electro Mechanical Systems) sensors (e.g., accelerometers, gyroscopes, GPSs), Wi-Fi, bluetooth, etc., have been widely adopted in the following areas: smart healthcare, intelligent transportation systems, smart buildings, and earthquake early warning systems [2,3,4,5]. In particular, the MyShake project [6] leverages mobile technologies to develop an earthquake early warning system that combines a seismic method and a machine learning (ML) technology. The system is installed on a volunteer’s smartphone and then detects earthquakes using an Artificial Neural Network (ANN). It is the first global earthquake detection system using a smartphone and machine learning technique.

Based on the available literature, we can divide IoT-based earthquake detection into two parts by applicability. A mobile-based earthquake early warning system uses low-cost MEMS sensors in a smartphone or an IoT device as a seismic sensor in a dynamic environment, while the stationary sensor-based early warning system uses a dedicated device as a seismic sensor in static (i.e., fixed) environment. The non-earthquake data in a static environment include internal and external noises. The source of internal noises mainly come from a sensor in which an accelerometer continuously captures some vibratory signals. The external noises come from outside of a sensor because of constructions, heavy-traffic roads, etc., near the installed sensor. In a dynamic environment, the variety of human activities become a major part of the non-earthquake data, which significantly affects the system performance, and thus the earthquake detection task using a low-cost sensor is very challenging. In this environment, training a machine learning algorithm is critical because of the activities whose frequency and amplitude patterns look like earthquakes.

In traditional earthquake early warning systems, because acceleration data recorded from seismic sensors installed nationwide are sent to a centralized server for earthquake detection, network and processing delays are inevitable. Because there are a few seconds between a P-wave and an S-wave (e.g., 10 s [7]) depending on the distance from an hypocenter [8,9], to reduce the blind area of earthquake early warning, on-site or standalone earthquake detection devices have been recently introduced [10,11,24]. However, because of the real-time processing requirement and resource constraints of a detection device, heavy computational methods and deep neural networks cannot be applied at the sensor side. Nevertheless, the final detection can be performed at the server-side through advanced detection algorithms, a simple detection algorithm with a few features that require light computations at a client-side to complete the detection procedure as soon as possible is required. Because an earthquake detection device can be operated in either a static or a dynamic environment, trivial statistical amplitude and frequency features are not suitable for such environments.

As a result, our focus is to improve a machine learning model for an earthquake alert device that we developed in our prior work to detect earthquakes in static and dynamic environments [10,11]. The device not only detects an earthquake but also sends an alert with earthquake response information to nearby smart devices such as smartphones, smart TVs, etc. As the device operates independently, without any Internet connection or collaborations with other alert devices, it needs a highly accurate earthquake detection algorithm. Because traditional methods to detect earthquakes such as STA/LTA have high false alarm rates, it is risky to use only one earthquake detection method for a standalone device. Thus, we use both traditional earthquake detection methods and emerging technologies together to decrease the chance of false alarms and increase the overall earthquake detection ability. In this article, we systematically compare different earthquake features and datasets representing static and dynamic environments for the earthquake alert device, and then, based on our experimental results, we propose a new earthquake detection model that can be used in both static and dynamic environments.

The rest of the article is structured as follows. Section 2 introduces our prior work and compares relevant research efforts. Section 3 explains the methodology used in the proposed work, while Section 4 discusses (in detail) the experimental work done. Finally, Section 5 concludes this article.

## 2. Prior Work and Related Work

In this section, we introduce our prior work for earthquake detection using emerging technologies and then compare our work with related research efforts.

### 2.1. Prior Work

**Figure 1:**
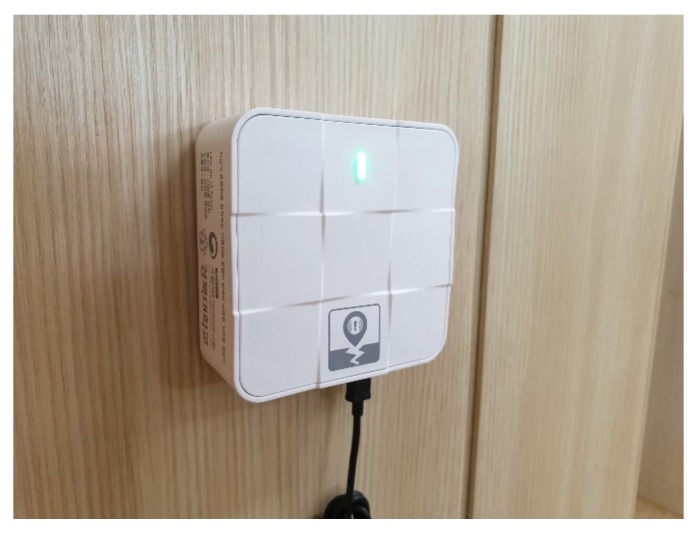
The developed earthquake alert device.

In our prior work [10,11], we developed an earthquake alert device that includes a 32 bit processor, Wi-Fi, bluetooth, a buzzer, an LED light, etc as shown in Figure 1; its hardware system is described in Table 1. To detect an earthquake, the earthquake alert device uses a machine-learning-based algorithm and then sends out an alert message to nearby devices such as smartphones, smart watches, AI speakers and home automation devices, using Bluetooth or Wi-Fi.

**Figure 2:**
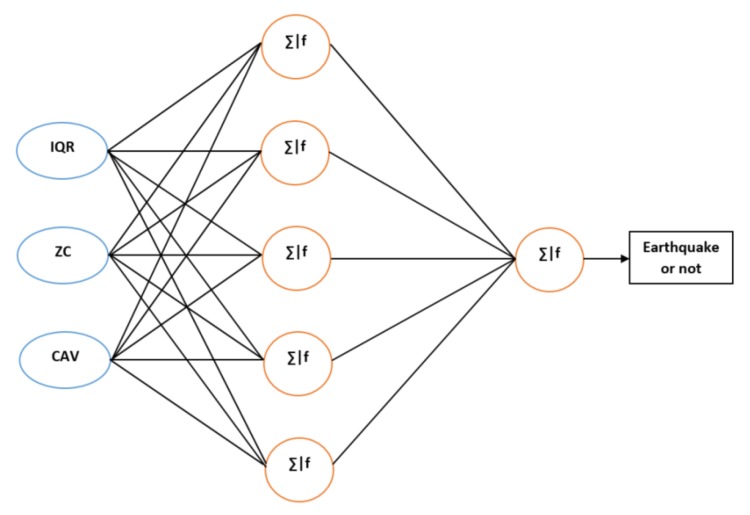
The ANN model with three inputs with one hidden layer.

The detection algorithm that we developed for the earthquake alert device is based on an artificial neural network (Artificial Neural Network) [12], which is a simple machine learning technique widely used in the last several decades. The used ANN model has three neurons in the input layer, five neurons in the hidden layer, and one neuron in the output layer as shown in Figure 2.

The detection algorithm consist of four phases including feature extraction, pre-processing, training, and testing of a machine learning model. To detect earthquakes, we use three features including inter-quartile range (IQR), zero crossing rate (ZC), and cumulative absolute velocity (CAV), which are the same features used in MyShake [6]. IQR is the amplitude between 25% and 75% of the acceleration vector sum. ZC is a frequency measure that indicates the number of time that the signal changes its sign. CAV is a cumulative measure of amplitude of the vector sum of three component acceleration. Then, we use 2 s of a sliding window with a 1.5 s overlap window on the acceleration data to calculate these three features in real-time.

After the extensive experiments, we installed devices in 29 locations of three different cities and operated them for two months. Even though the model showed a high accuracy of over 95% in our experiments, we found a few false alarms throughout the real operation. Therefore, in this article, we carefully assess the performance of the earthquake detection model and test its added features to determine the best features for earthquake detection in our operational environments. In the rest of the article, we present our efforts on the development of various features for earthquake detection and experimental results.

### 2.2. Related Work

Various monitoring systems leveraging mobile technologies have been proposed, such as eWatch, smartphones, and MEMS [13]. In particular, extensive research has been done on wearable IoT in healthcare. For example, eWatch [14] is an online activity recognition system that embeds four different sensors, i.e., an accelerometer, alight sensor, microphone, and thermometer. The system is very responsive and needs no wireless communication. Similarly, Kao et al. [15] have used a tri-axial accelerometer in a portable device that can be placed on the user’s dominant wrist to detect human activities, such as running, working, swinging, brushing teeth, knocking, walking, and hitting. The accelerometer of a smartphone has also been used for human activity recognition, such as walking, running, walking (fast and slow), climbing stairs (up and down), and excercising aerobatics [16]. In the literature, there are many applications that used a sensor-based monitoring system; however, these are beyond the scope of this article. Instead, we deal with the binary classification problem, and our goal is to detect earthquakes from the accelerometer data in which the rest of the data is the non-earthquake class, whether that includes human activity or noise.

Traditional seismic detection involves computational methods such as Short-Term Average/Long-Term Average(STA/LTA), cross-correlation, and template matching [17,18,19,20,21]. These methods are useful but have certain limitations. For example, STA/LTA can detect earthquakes without prior knowledge of the event but can also produce false positives when the situation is more challenging, such as when it involves a low signal to noise ratio (SNR), overlapping events, and some cultural noise. Similarly, cross-correlation detects earthquake signals but is computationally expensive, while template-based matching is a powerful computational method but requires prior information. The above methods are mostly operational in the central system. Moreover, the computational methods do not exhibit any intelligent behavior and operate only on the fixed threshold values.

Recently, there have been research efforts to use MEMS-based sensors as seismic sensors due to their low computational power and cost. Specifically, the NetQuakes project developed by the United States Geological Survey (USGS) installed MEMS sensors around the world but mostly in California [22] and began to collect seismic data from them. Similarly, the following projects developed around the world use MEMS sensors; Home Seismometer Network (HSN) developed by Japan Meteorological Agency (JMA), Palert system developed by NTU(National University Taiwan), Community Seismic Network and Quake-catcher Network (QCN) developed by California Institute of Technology and Standford University, respectively [23,24,25,26].

IoT systems for public safety are widely adopted, where the intelligence behavior of such sensors as MyShake, which combines machine learning with traditional STA/LTA algorithm, limit or exclude human intervention [27]. To our knowledge, this is the first globally used smartphone-based earthquake early warning system used in a dynamic environment. Besides, deep learning approaches have also been adopted to detect earthquakes offline or online at the server-side, such as searching seismic data, mining undetected earthquakes in the data archives, and finding the earthquake location [28,29]. In this article, our first goal is to improve the existing earthquake detection model’s performance in the static environment. The second goal is to evaluate the machine learning algorithms and feature sets (both existing and proposed) for sensor-side in the dynamic environment with a variety of human activities.

## 3. Proposed Methodology

This section will discuss the feature extraction and machine learning methodology. The proposed work follows the supervised machine learning methodology. The steps involved in our proposed methodology are feature extraction, preprocessing, training, testing, and validation.

### 3.1. Feature Extraction

In the context of ML-based earthquake detection, amplitude and frequency are the two key pieces of information among different statistics of the accelerometer signal. Therefore, based on these two statistics, we extracted features from *X*, *Y*, and *Z* components in the time and frequency domains. Time domain features include features used in MyShake and our proposed features. The MyShake features are the following.

- IQR (Interquartile Range): IQR is the interquartile range Q3–Q1 of the 3 component vector sum VS;
  (1) $$VS=\sqrt{X^{2}+Y^{2}+Z^{2}}$$
  where *X*, *Y*, and *Z* are the acceleration components.
- CAV (Cumulative Absolute Velocity): CAV feature is the cumulative measure of the VS in the time window and is calculated as
  (2) $$CAV=\int_{0}^{s}\left|VS\left(t\right)\right|dt$$
  where *s* is the total time period of the feature window in seconds, and *t* is the time. In this work, we used a two-second feature window.
- ZC (Zero-Crossing): ZC is the maximum zero-crossing rate of *X*, *Y*, and *Z* component and the zero-crossing rate of component *X* can be calculated as:
  (3) $$ZC_{X}=\frac{1}{N-1}\sum_{t=1}^{N-1}1_{R_{<0}}\left(X_{t}X_{t-1}\right)$$
  where *N* is the total length of the signal *X* and $1_{R_{<0}}$ is indicator function.

IQR and CAV are the amplitude features, while ZC is the frequency feature, and these are proposed in [6,30]. These features detect earthquakes and can discriminate non-earthquake data, but through exhaustive experimental evaluations and also its implementation in the static environment as given in our previous work, we found that in a noisy environment (noisy sensors or external events), its performance can be degraded. Moreover, a dynamic environment—in which the variety of human activities that include some challenging activities whose signal patterns are similar earthquake patterns—can also degrade the performance of the model trained on these features. We observed that among the three features, zero-crossing is more sensitive to noise and creates false alarms even if there is wavering involving only one component. This is due to the fact that it counts the feature value for each component and then selects the maximum one. Hence, if there is a count at only one component, then it will select that value and discard the zero-crossing information of the other two components. We observe that earthquake motion has a zero-crossing rate at more than one component simultaneously, while other data—particularly noise data—have zero-crossing rates at only one component most of the time, as given in Table 2. Two-second feature window with a 1-second sliding window is used to count ZC in both earthquake data and noise data, where, for the earthquake data, we selected 3 s of the strongest portion of the earthquake. Further details about datasets are given in the results section.

This sensitivity issue not only affects the performance of the machine learning model in a dynamic environment (when the sensors are assumed to be smartphones used in daily life) but also affects the model performance while in a fixed-sensors environment. Therefore, to overcome this issue, we tested different variations and statistical features of the amplitude and frequency characteristics of the signal. After extensive experiments, we proposed some variants of the zero-crossings, which are the following.

- Max ZC: Counts for that component whose maximum absolute amplitude value is greater than the other two components when there is more than one zero-crossings at a particular time t. Otherwise, it will behave like the ZC feature.
- Min ZC: Counts for the minimum one, which has lowest absolute amplitude value among the three, if there are zero-crossings in more than one component.
- Max Non ZC: This feature counts the maximum absolute amplitude component for non-zero-crossings when there is more than one non-zero-crossings simultaneously at a particular time.

These features are also based on the frequency and amplitude information of the signal; however, these are more specific and consider the other statistics, like multi-component zero-crossings and the frequency information, when there is no zero-crossing. The non-zero-crossing statistic is also important, because if the occurrences of ZC indicate the probability of an earthquake situation, then this feature indicates the probability of a normal situation. Similarly, the multi-component property of these features is also helpful to discriminate human activities from earthquake samples more efficiently.

**Figure 3:**
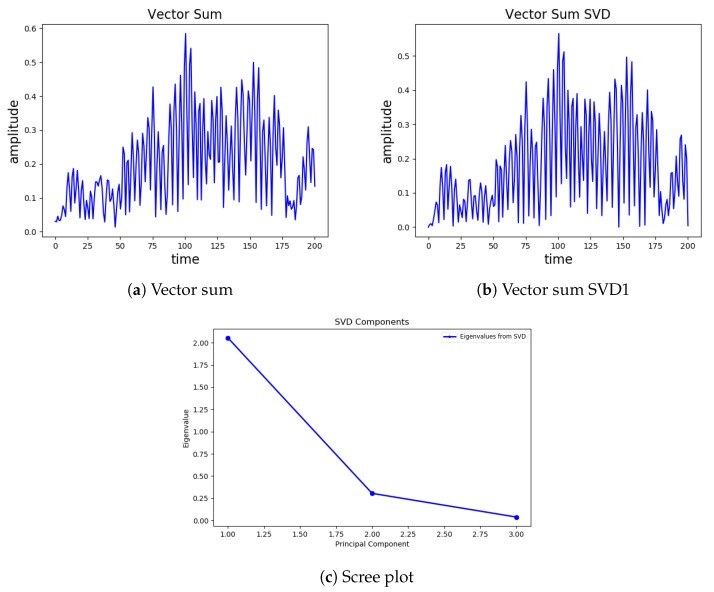
A two-second window of the strongest portion of the earthquake; (**a**) The vector sum of X, Y, and Z; (**b**) vector sum of the primary vector of SVD (center); (**c**) scree plot of the three components.

Apart from the proposed features, we also tested features from the frequency domain, i.e., FFT (Fast Fourier Transform) [31]. In order to consider only one component of FFT, we used a Singular Value Decomposition (SVD) method to decompose multi-dimensional data into one dimension [32]. The SVD of an accelerometer matrix *A* of three components, X, Y, and Z.

(4) $$A=USV^{T}$$

where, *A* is an M x N matrix, where M represents two-second points, i.e., 200, and N is 3. SVD provides three new vectors $U_{MxM},\;S_{MxN},\;and\;V_{NxN}^{T}$, which, if linearly combined, give back the approximated original vector; where *U* is the set of singular vectors with singular values in vector *S*, $V^{T}$ is the primary direction. The new vectors are ordered, and the first vector explains most of the original acceleration amplitude and frequency information, as shown in Figure 3. Figure 3a depict almost the same structure; therefore, we select the first vector as a primary vector U[:,0] from the given SVD’s, along with the first value S[0] of *S*, which is a scaling factor (give amplitude information of the given vector). We extracted the following three additional features.

**Figure 4:**
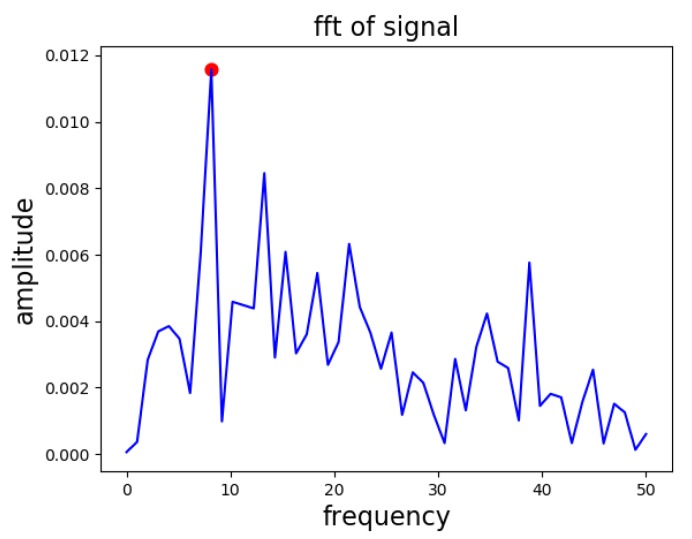
FFT of the primary vector of the SVD extracted from the two-second strongest portion of the earthquake window.

- FFT: FFT of the given vector U[:,0] is calculated, and we selected the frequency bin as a frequency feature that has the peak amplitude, as shown in Figure 4.
- SVD_Scale: The S[0] is considered the average amplitude feature.
- SVD_ZC: We also computed the zero-crossing rate of the primary vector i.e., U[:,0].

We also analyzed the tsfresh [33], a time series feature-extraction python package for searching the computationally low and effective features such as c3, cid-ce, entropy, mean, and count-above-mean, etc. However, the feature space visualization was not more promising than the abovementioned features. Therefore, we selected only the above features for model training and testing.

### 3.2. Pre-Processing

**Figure 5:**
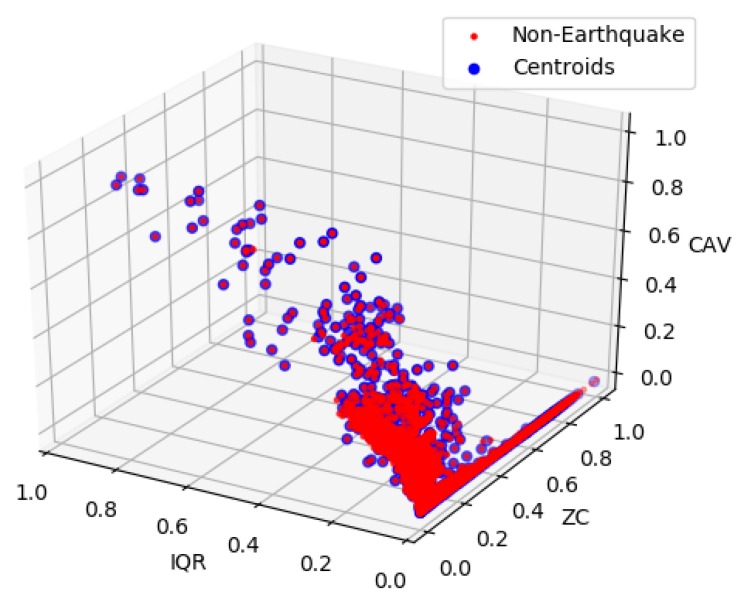
Centroids of the non-earthquake data (noise with some human activity).

In our methodology, the pre-processing involved balancing the dataset and scaling the features to range from 0 to 1. Balancing is required because the imbalanced datasets greatly affect the performance of the machine learning model [34]. In our case, the non-earthquake dataset (noise and human activities) is much larger than the earthquake dataset. Hence, we used the K-mean clustering algorithm to balance the non-earthquake dataset [35]. Using the K-Mean, clusters of the non-earthquake data are created according to the total number of earthquake data points, and we used centroids of the clusters to represent the non-earthquake data. As shown in Figure 5, centroids represent the original data points in the IQR, ZC, and CAV feature space.

Moreover, to improve the prediction performance and decrease the training time of the model, we also scaled data point *d* to the range of 0 to 1 using the min-max scaler as follows:

(5) $$d_{scaled}=\frac{d-d_{min}}{d_{max}-d_{min}}$$

### 3.3. Machine Learning Model

**Figure 6:**
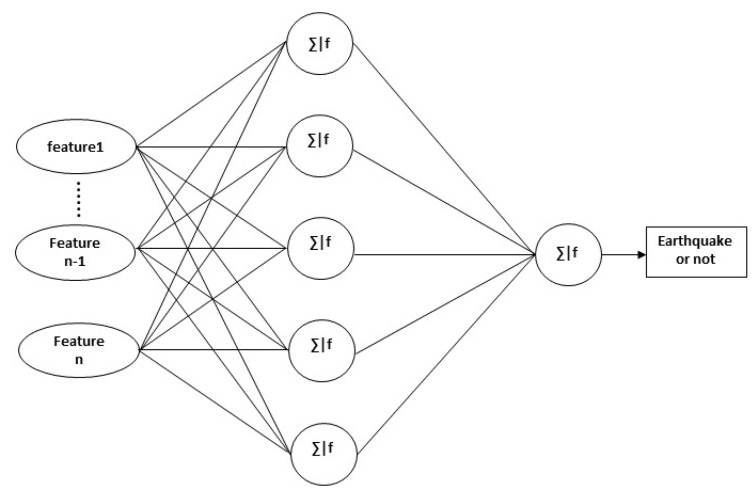
The general structure of the ANN model.

The ANN (Artificial Neural Network) algorithm is designed to accomplish the detection task using both the existing and proposed features [12]. We used an X, 5, 1 layer network architecture for the training and testing of the ANN algorithm, as shown in Figure 6, where X is the number of features input to the model. We kept the same five nodes of the hidden layer as proposed in [6], because the number of features input to the model is 3, 4, or 5, and through experimental results, the 5-node hidden layer is still good for the given number of features. For training the models, we used a multi-layer perceptron (MLP) with the stochastic gradient descent solver [36,37,38]. For the hidden layer and output layer, the inputs from the previous layer to each node will be first summed and then fed into an activation function as follows:

(6) $$y=\phi (\overset{n}{\underset{i=1}{\sum }}w_{i}d_{i}+b)$$

Here, *w* denotes the weights vector, *d* is the input vector, *b* is the bias, *y* is the output of the given node, and ϕ is the non-linear activation function. The logistic sigmoid function is used as the activation function for hidden and output layers, which is defined on input *d* as

(7) $$\phi \left(d\right)=\frac{1}{1+e^{-d}}$$

## 4. Results and Discussion

To obtain a comprehensive comparison, we compared the proposed features with the existing features in both the static and dynamic environments. Accordingly, we trained ANN models with different non-earthquake datasets to distinguish the environments.

### 4.1. Dataset

The dataset that we used for training and testing the ANN models contains two classes of label data. One class of data is the time series earthquake dataset, which was download from the National Research Institute of Earth Science and Disaster Prevention (NIED) and USGS (United States Geological Survey) database [39,40]. A total of 385 earthquakes events with magnitudes ranging from 4 to 8, recorded between April 2009 and May 2019, were selected from the NIED database. Moreover, 120 stations’ data of three earthquakes, i.e., Tottori (2000) (magnitude 6.61), Niigata (2004) (magnitude 6.63) and Chuetsuoki (2007) (magnitude 6.8) were downloaded from the USGS database. The NIED earthquake data were pre-processed and converted into the unit (g). The sampling rate of all the earthquake data is 100 Hz. The data are presented in three columns titled EW, NS, and UD, respectively, where EW (East-West) and NS (North-South) are horizontal components, and UD (Up-Down) is a vertical component.

The second class of data is the time series of non-earthquake dataset recorded on mobile phones for several hours. In the experiments, we used two types of non-earthquake data, i.e., human activity data and noise data. Human activity data includes such activities as bicycle, bus, and car (in hand) riding, jump rope, running (hand, pocket), desk shaking (while mobile on top), climbing stairs (up-down) (bag, hand, pocket), walking (bag, hand, pocket), standing still, and working. Contrarily, noise data contain floor noises (e.g., different degrees of elevations) and machinery noises. These noise data are the external source data; and hence, to include sensor noise data, we also include the tail data of earthquake signals.

The models’ generalization characteristics are validated on the third dataset, which is earthquake data collected during shake table tests using different accelerometers (i.e., ADXL355 [41], LIS3DHH [42], MPU9250 [43], and MMA8452 [44]), which have different HW specifications and costs. Sensors were placed on the shake table located in Pusan National University to record two realistic earthquakes including Pohang [45] and El Centro [46], and we collected acceleration data from such low-cost accelerometers.

### 4.2. Performance Metrices

**Figure 7:**
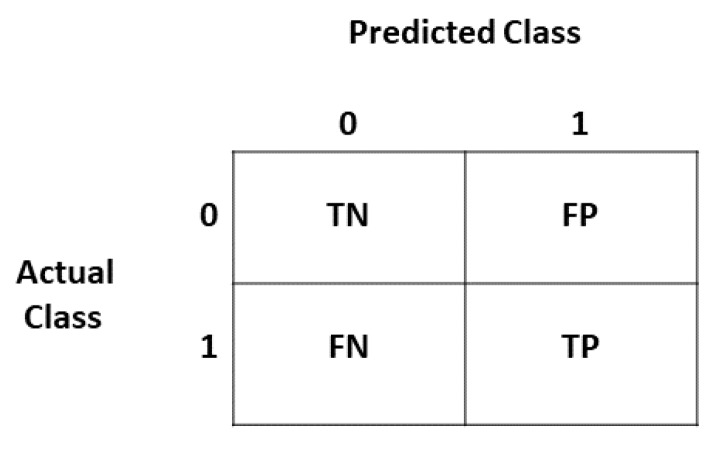
Confusion Matrix.

Different machine learning algorithms are evaluated with different performance matrices. The classification performance metrics are based on the confusion matrix [47], which gives a table of TP (True Positive), TN (True Negative), FP(False Positive), and FN (False Negative) as shown in Figure 7.

Common performance measures of the classification such as accuracy, precision, and recall, are calculated from the confusion matrix. Accuracy is computed as

$$\mathrm{Accuracy}=\frac{TP+TN}{TP+FP+FN+TN}$$

Accuracy is the ratio of predictive observations to the total observations; it is an intuitive measure to show the overall performance of the model.

Precision is computed as

$$\mathrm{Precision}=\frac{TP}{TP+FP}$$

The precision measure determines how accurate the model is out of those predictive positives. In other words, how many of them are actually (correctly) positive among all predictive positives. High precision means a low false-positive rate.

Recall is computed as

$$\mathrm{Recall}=\frac{TP}{TP+FN}$$

Recall determines the sensitivity of the model in terms of how many times it detects earthquakes from all the instances of the earthquakes.

The F1 score is a single score of precision and recall which is the harmonic mean of both. It takes both false positive and false negatives into account.

$$F1\;\mathrm{score}=\frac{2}{1/precision+1/recall}$$

Finally, the classification model performance false and true positive rates can be visualized through a receiver operating characteristics (ROC) curve [48].

### 4.3. Evaluation

The evaluation is done in static and dynamic environments. In the static environment, the sensors are fixed (stationary) and, therefore, training a model with varieties of human activities is not required. Still, to train the model properly for the static environment, we used some instances of human activities like walking and waiting. This is because the model converges too quickly in the presence of only noisy data and thus cannot learn the underlying patterns of the data, especially earthquake patterns. We evaluated models based on different features and then, for the dynamic environment, we tested the model that showed the best results in the static environment to evaluate its full implementation applicability.

### 4.4. Static Environment

During the model evaluation in the static environment, we used a combination of different features discussed above. We trained the model using amplitude features combined with frequency features. Here, from the sets of different models, we will discuss six models, beginning with the existing MyShake model, i.e., IQR, ZC, and CAV (Model 1). The remaining five models with feature sets are given below.

- Model 2: IQR, FFT, CAV
- Model 3: IQR, ZC, FFT, CAV
- Model 4: IQR, SVD_ZC, CAV
- Model 5: IQR, ZC, CAV, SVD_Scale
- Model 6: IQR, CAV, MAX_ZC, MIN_ZC, MAX_NON_ZC

Through the experimental search, the five-nodes hidden-layer structure was used for training all the models, and the input layer nodes were determined according to the feature set. Table 3 provides details of the dataset used for training and testing the models in the static environment.

**Figure 8:**
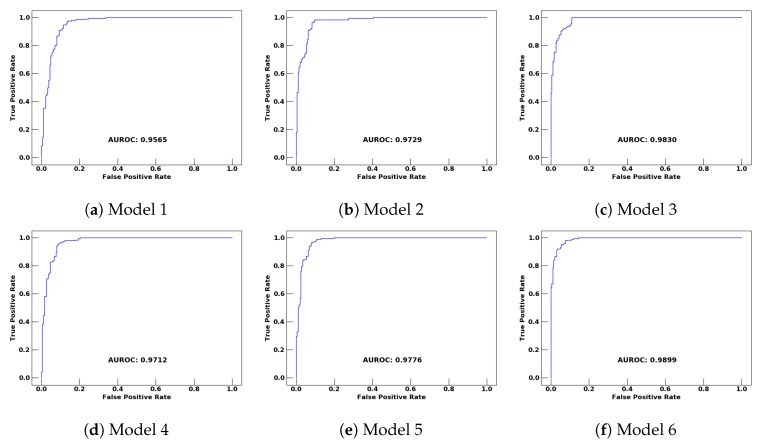
ROC curves of the models on 20% test data.

For training and testing the models, we split the data (earthquake and centroids of the non-earthquake) into 80% and 20%, respectively. In terms of testing the models, we first tested each model on the remaining 20% of the centroids (experiment 1), and then, in the second experiment (experiment 2), the models were tested on the original data (all the instances of the earthquake and non-earthquake class). For the receiving operating characteristics curves of the ANN models, 20% of the remaining data are shown in Figure 8. All the models showed good results, where Model 6 shows the high AUROC of 0.9899 and rapid climb, which is close to the ideal case.

Despite the fact that all the models showed good results, other performance measures of the models should also be considered. Table 4 gives the performance score of the models on the two test datasets; the first test dataset is the remaining 20% of the centroids data and the second test dataset is the original non-earthquake data. Among all the models, Model 6 successfully classified the earthquake instances in both experiments (i.e., centroids and original data) with high accuracy, F1 score, and a low number of false positives. The false positive in the second experiment is comparatively high as compared to experiment 1 because there are more data points for a particular non-earthquake category with variations in the data. Still, the accuracy score of both experiments was very good. The accuracy and recall of the second experiment are slightly better than experiment 1, which indicates that the model is also trained well for unseen data to deal with the over-fitting problem. As a single frequency feature, FFT standalone cannot provide assistance to the model, as shown in the results of Model 2. However, with the ZC feature, it showed some improvement in the performance of Model 1, as seen in the Model 3 row. The new features of ZC_SVD and SVD_Scale can be used as a frequency feature and amplitude feature as suggested by Models 4 and 5, respectively.

The FP counts of Model 1 indicate that the model is sensitive to the noise due to the frequency feature of ZC, as discussed earlier. However, Model 6 has three different statistics for the frequency information, which gives more information to the machine learning model and contributes to the improved results of the model. It has been observed that, for the amplitude information, IQR and CAV are still good features, but the frequency feature is the most sensitive one since it is not only affected by the noise but also by the difference of the sampling rates.

#### Models Validation

As described in the dataset section, to further validate and compare these models on the unseen data, we used Pohang and El Centro earthquakes data. Here, we used 100% and 50% scale of both the earthquakes, where a 100% of Pohang and El Centro respectively represents approximate magnitudes of 5.4 and 6.9 earthquakes. Similarly, the 50% scale earthquake data represents moderate and low amplitude earthquake data, which allows us to evaluate the model performance on these low scale data.

During each test, these 100% and 50% scale data of both the earthquakes are input into the trained models. The duration of each earthquake data is 70–80 s, and the features are extracted from each sensor’s data according to the model feature set; for example, for Model 1, we extracted IQR, ZC, and CAV features. A two-second sliding window with a one-second overlap window was used on the raw acceleration to extract features.

**Figure 9:**
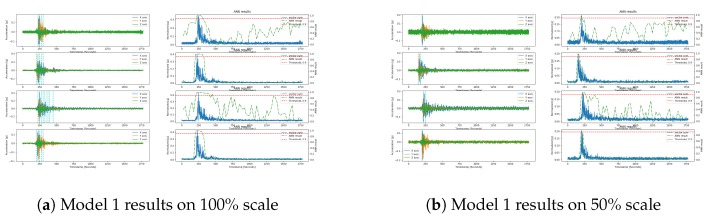

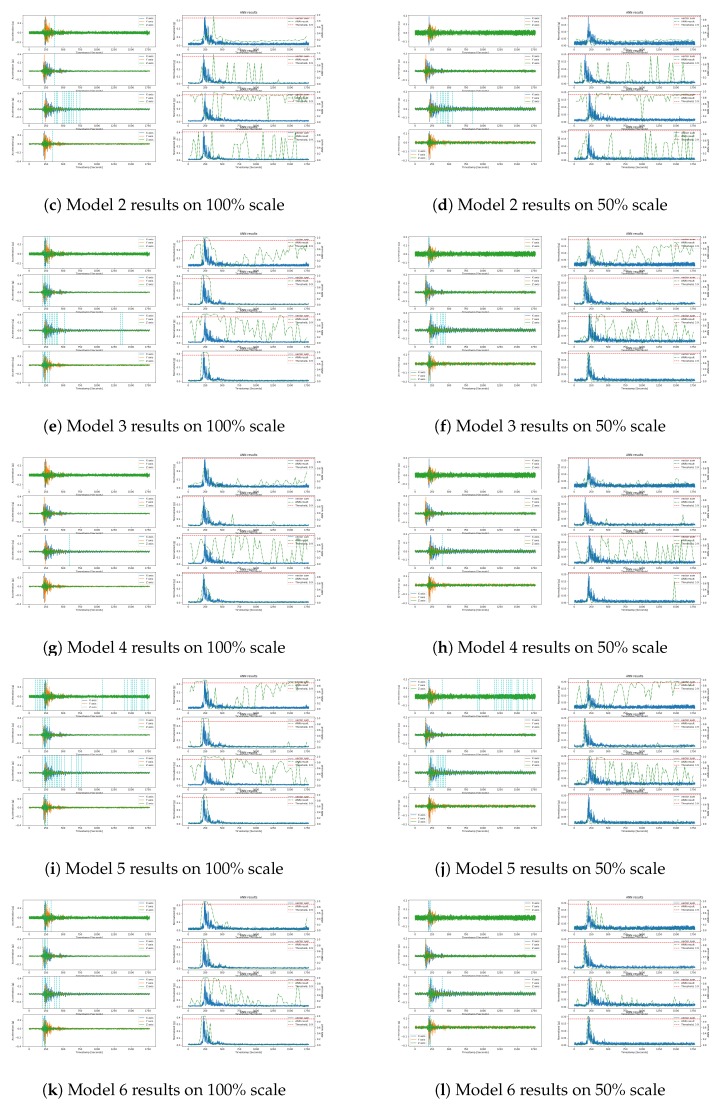
Models’ test results on the Pohang earthquake at a scale of 100% (left) and 50% (right); the results are ordered from top to bottom for each subfigure’s sensor, namely ADXL355, LIS3DHH, MMA8452, and MPU9250.

**Figure 10:**
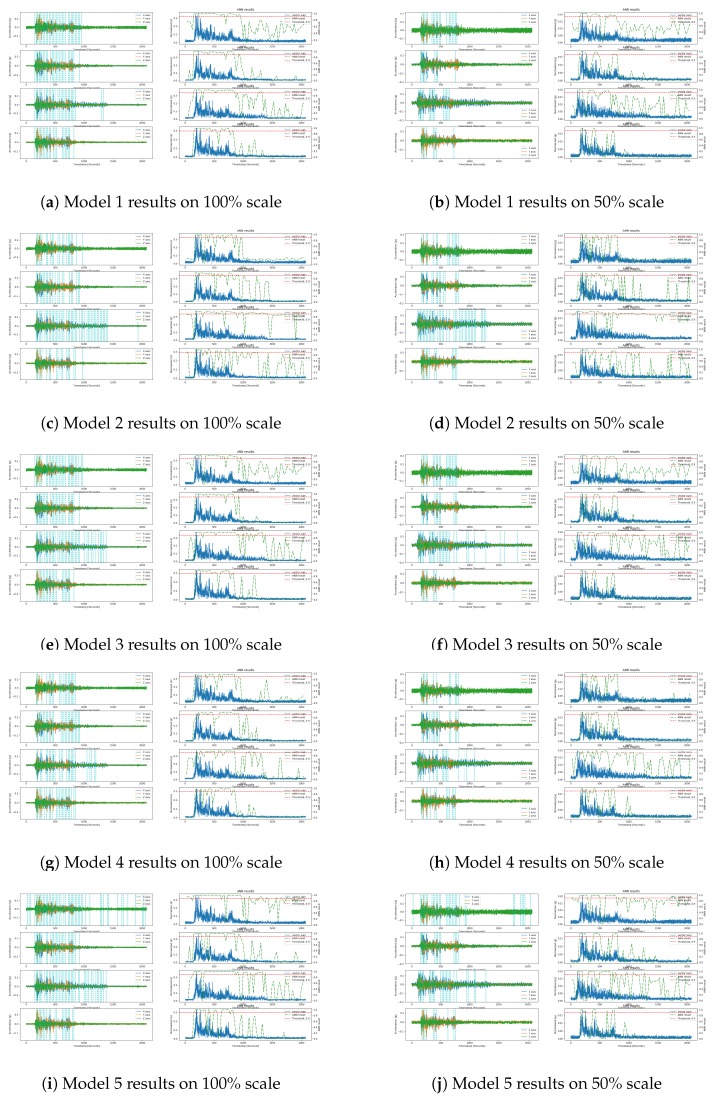

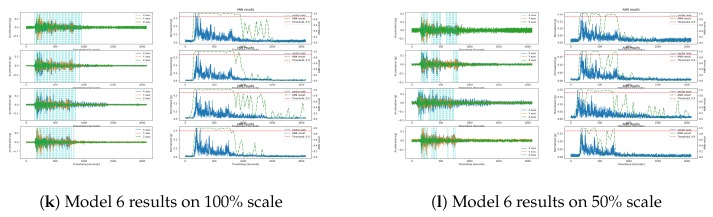
Models’ test results on the El Centro earthquake at a scale of 100% (left) and 50% (right); the results are ordered from top to bottom for each subfigure’s sensor, namely ADXL355, LIS3DHH, MMA8452, and MPU9250.

The test results of the models on the Pohang and El Centro data are given in Figure 9 and Figure 10, respectively. Figure 9a shows the results of the detection process for Model 1 (right panel) on the normalized vector sum of three axes, along with the raw acceleration (left panel) of X, Y and Z components of the Pohang earthquake on the 100% scale. The sampling rate of the signal is 25 Hz and the threshold value is kept at 0.9 for the ANN models to detect earthquake triggers. The reason for choosing this threshold value is to decrease the FP rate of the model in the system implementation. As given in Table 5, FP counts of the models’ on the original test data decreased, as a result, the precision of the models increased but the recall is also decreased. Here, we can see that the F1 score describes the overall performance of the models, and again Model 6’s F1 score is high compared to other models when the threshold value is set to 0.9. The cyan vertical line in the left panel represents the earthquake trigger when the ANN probability (wavy green line in the right panel) meets the threshold value (red line in the right panel).

Model 1 detected earthquakes in both the 100% and 50% scales of the Pohang earthquakes, and did so at peak acceleration with very low false alarms, as shown in Figure 9a,b. The ANN probability graph shows very smooth and stable probabilities in the LIS3DHH and MPU9250 cases during the non-earthquake portion, while the other two cases (i.e., ADXL355 and MMA8452 results, show high peaks due to sensor noise). In our previous publications [10,11], we observed that ADXL355 has noise on one component while MMA8452 has noise on all the components, which confused the model due to the zero-crossing feature. Model 1 produced similar results for the El Centro earthquake data across different scales, as shown in Figure 10a,b. In the El Centro case, as we can see its signal pattern is different from the Pohang pattern, which resulted in more false triggers generated by Model 1.

Compared to Model 1, the proposed Model 6 showed promising results on the validation data of Pohang and El Centro earthquakes of different scales and sensors. as shown in Figure 9k,l and Figure 10k,l. The proposed Model 6 shows better performance on the accelerometer sensor data, where Model 1 produced high peaks in the probability graphs. Further, in the case of the ADXL355 accelerometer sensor, Model 6 was able to with the problem of noise on one component due to its extensive information about zero-crossing frequency. Multi-component noises can be a challenge for model performance, as the MMA8452 case reveals.

Model 2 shows poor results due to the lack of frequency information in the time domain, as shown in Figure 9c,d and Figure 10c,d. Despite having tested the FFT feature to provide the frequency information, through the experimental work, we observed that a single FFT feature is not enough to train the model. Then, although combine with the ZC feature it showed slightly better results than Model 1 in 20% test, the results of the Pohang and El Centro test data were poorer than those of Model 1, as shown in Figure 9e,f and Figure 10e,f. Similarly, the results of model 5 were also insignificant, as shown in Figure 9i,j and Figure 10i,j.

To Summarize, Models 1 and 6 show outstanding and consistent results in the unseen data, whereas other models show different performance results in different experimental scenarios. Therefore, we chose to test Models 1 and 6 in a dynamic environment.

### 4.5. Dynamic Environment

In the dynamic environment, we evaluate Model 6, which showed very good results as compared to other models, including the model used in MyShake. Therefore, to evaluate the model with the proposed features in the dynamic environment, we considered all the human activities recorded on smartphones for several hours. Due to the increase of non-earthquake data, we also include more earthquake data to keep the balance between earthquakes and non-earthquakes and then test the model performance on the larger datasets. The trained model is referred to as Dyn-model 6, and to compare the model with the state-of-the-art Myshake features, we also train Model 1 using the dynamic dataset and referred to it as Dyn-model 1. Table 6 provides details of the dataset used for training the models in the dynamic environment.

We perform the same methodology used for the static data; that is, we first extracted features from the non-earthquake data then calculated centroids equal to the earthquake data points. To train the model, we split the data (earthquake and centroids) into 80% and 20% for training and testing, respectively. The best test results from a number of experiments are given in Table 7. We can see that the accuracy of the Dyn-model 6 on the original data is higher than that of the centroids data, whereas the Dyn-model 6 accuracy on 20% is similar to its accuracy in the static test (i.e., approximately 94%). However, its accuracy on the original data in the dynamic test is lower than the static test due to the variation in the non-earthquake data. In the original data, the Dyn-model 6 falsely detects 1804 non-earthquake instances as earthquakes. We further investigate the Dyn-model 6 results on each activity and found that the FP of the Dyn-model 6 was mostly produced due to human activities such as bus riding, desk shaking, and walking, with the accuracy of 90.3%, 93.23%, and 91.65%, respectively.

Like Dyn-model 6, Dyn-model 1 accuracy was also decreased due to the activities that can result in earthquake-like signals. Moreover, Dyn-model 6 results are better than those of Dyn-model 1 results due to the increasing frequency features and different recalculation of ZC to make it conditional to the maximum amplitude.

#### Model Validation

**Figure 11:**
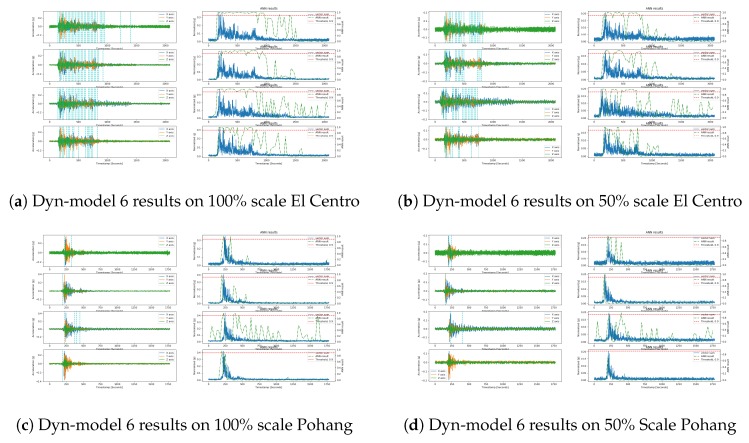
Dyn-model 6 test results on the El Centro earthquake of scale 100% (left-top) and scale 50% (right-top); Model test results on the Pohang earthquake of scale 100% (left-bottom) and scale 50% (right-bottom); the results are ordered from top to bottom for each subfigure’s sensor, namely ADXL355, LIS3DHH, MMA8452, and MPU9250.

Similar to the validation for the static environmental model, we validated Dyn-model 6 on the unseen earthquake datasets generated during shake table tests on different sensors, i.e., El Centro and Pohang, as shown in Figure 11. This was conducted because our ultimate goal was to see whether the trained model can detect earthquakes when the device is in a steady-state, as compared to the proposed Dyn-model 6, which was trained for the static environment and then retrained for the dynamic environment. The newly trained Dyn-model 6 showed almost the same detection results, but with fewer earthquake detection triggers during the earthquake windows for both the scales of the El Centro and Pohang earthquakes. The proposed Dyn-model 6 also detected some false triggers as can be shown in Figure 11a. In particular, the proposed Dyn-model 6, when trained with dynamic data, showed far fewer detection triggers on the Pohang earthquakes; rather than detecting the earthquake, it only showed a peak below the given threshold of 0.9 for the data recorded on the MPU9250 scale 50%, as shown in Figure 11d. These results indicate that the model learned differently, and the challenging non-earthquake data can affect the model performance, which can result in a false alarm, whether false positive or false negative.

**Figure 12:**
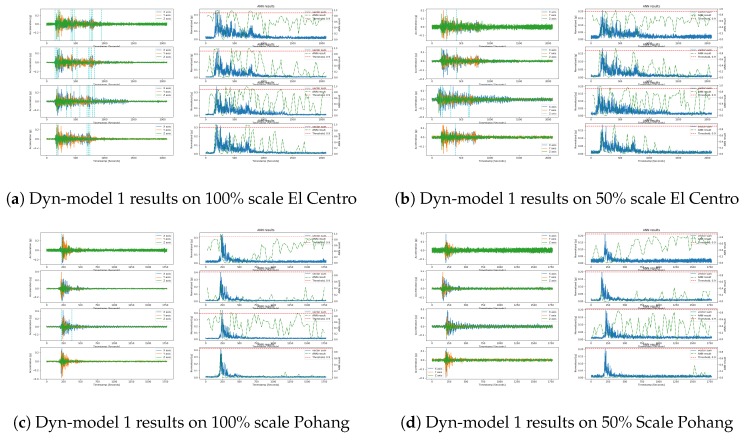
Dyn-model 1 test results on the El Centro earthquake of scale 100% (left-top) and scale 50% (right-top); Model test results on the Pohang earthquake of scale 100% (left-bottom) and scale 50% (right-bottom); the results are ordered from top to bottom for each subfigure’s sensor, namely ADXL355, LIS3DHH, MMA8452, and MPU9250.

The validation results of Dyn-model 1 on the Pohang and El Centro datasets are given in Figure 12. This time, Dyn-model 1 results are not as promising as those of the static environment in all the windows. In particular, it failed to detect the earthquake in the Pohang 50% scale. It showed the same probability peaks in the ADXL355 and MMA8452 sensors in all the windows, which support our claim discussed with regard to the static environment. Moreover, compared to the proposed model that is Dyn-model 6, again the performance of the existing model that is Dyn-model 1 is below in the dynamic environment too.

### 4.6. Threats to Validity

The experimental results are subject to the following validity threats. Even though we deal with heterogeneous data recorded on different sensors, the models were trained on the data which were mostly recorded on the seismic sensors but we used low-cost accelerometers for validating their performance. Therefore, the experimental result may be different if the models are properly trained using earthquake data recorded on low-cost accelerometers. Furthermore, the datasets that we collected have different sampling rates. For instance, the models were trained with the earthquake data at a 100 Hz sampling rate and different sampling rate of non-earthquake data ranging from 50 to 100 Hz. Also, the sampling rate for the validation was 25 Hz. Despite above validity threats, the model showed outstanding performances, but the accuracy measurements may vary for different datasets and sampling rates.

## 5. Conclusions

In this article, we categorized seismic detection mechanisms into the static and dynamic environments and then evaluated different features using the ANN model in the static environment, which include new features and the existing features used in previous studies. Based on the experimental results performed in the static environment produced, the proposed features demonstrated more improved results than the existing features. For the dynamic environment, we used the same model tested for the static environment and then trained it with different datasets, which included various human activities. The selected model showed promising results with a lower possibility of false alarms than other models. As a result, our approach can be used for both a static and a dynamic environment without changing its model and features. As a future research direction, we will explore new features and models that require less computational power while maintaining a high detection ability against the challenging non-earthquake datasets.

## Figures and Tables

**Table 1:** Specifications of the developed earthquake alert device.

HW Component	Specification
**CPU**	1 GHz single-core ARMv6
**Memory**	512 MB
**Networks**	802.11n wireless LAN, Bluetooth 4.0
**Accelerometer**	LIS3DHH

**Table 2:** ZC count at only one component.

Class	Total Instances	One Comp: ZC Counts
**Earthquake**	730	14
**Noise**	19,813	18,489

**Table 3:** Dataset used for models training and testing in a static environment.

Class	Total Instances
**Earthquake**	1010
**Noise**	20,116
**Walk and Wait**	4000

**Table 4:** Performance evaluation of the ANN models on test data.

Model	Test Data	TP	TN	FP	FN	Accuracy	Precision	Recall	F1
**Model 1**	Centroids	203	164	20	17	90.84	91.03	92.27	91.65
	Original	962	22,556	1560	48	93.60	38.14	95.24	54.47
**Model 2**	Centroids	178	192	13	21	91.59	93.19	89.45	91.28
	Original	925	20,515	3601	85	85.33	20.44	91.59	33.18
**Model 3**	Centroids	210	171	21	2	94.31	90.91	99.06	94.81
	Original	993	22,797	1319	17	94.68	42.95	98.32	59.78
**Model 4**	Centroids	195	180	16	13	92.82	92.42	93.75	93.08
	Original	949	22,214	1902	61	92.19	33.29	93.96	49.16
**Model 5**	Centroids	193	185	21	5	93.54	90.19	97.47	93.69
	Original	984	22,537	1579	26	93.61	38.39	97.43	55.08
**Model 6**	Centroids	192	191	11	10	94.80	94.58	95.05	94.82
	Original	959	23,552	564	51	97.55	62.97	94.95	75.72

**Table 5:** Performance evaluation of the ANN models on the original test data using a 0.9 threshold value.

Model	TP	TN	FP	FN	Accuracy	Precision	Recall	F1
**Model 1**	698	23,987	129	312	98.24	84.40	69.11	75.99
**Model 2**	543	24,112	4	467	98.13	99.27	53.76	69.75
**Model 3**	815	23,536	580	195	96.92	58.42	80.69	67.76
**Model 4**	685	23,932	184	325	97.97	78.83	67.82	72.91
**Model 5**	781	23,814	302	229	97.89	72.11	77.33	74.63
**Model 6**	807	24,059	57	203	98.96	93.40	79.90	86.13

**Table 6:** Datasets used for model training and testing in a dynamic environment.

Class	Total Instances
**Earthquake**	2464
**Non-earthquake**	44,094

**Table 7:** Performance evaluation of the ANN models on the test data in the dynamic environment.

ANN Model	Test Data	TP	TN	FP	FN	Accuracy	Precision	Recall	F1
**Dyn-model 1**	Centroids	460	442	56	28	91.48	89.15	94.26	91.63
	Original	2315	39,935	4159	149	90.07	35.76	93.95	51.80
**Dyn-model 6**	Centroids	474	453	35	24	94.02	93.12	95.18	94.14
	Original	2336	42,290	1804	128	95.85	56.43	94.81	70.8
